# Combined Aerobic Exercise and Neurofeedback Lead to Improved Task-Relevant Intrinsic Network Synchrony

**DOI:** 10.3389/fnhum.2022.838614

**Published:** 2022-06-14

**Authors:** Saurabh Bhaskar Shaw, Yarden Levy, Allison Mizzi, Gabrielle Herman, Margaret C. McKinnon, Jennifer J. Heisz, Suzanna Becker

**Affiliations:** ^1^Department of Psychiatry, Western University, London, ON, Canada; ^2^Vector Institute for Artificial Intelligence, Toronto, ON, Canada; ^3^Homewood Research Institute, Guelph, ON, Canada; ^4^Department of Psychology Neuroscience & Behaviour, McMaster University, Hamilton, ON, Canada; ^5^Centre for Advanced Research in Experimental and Applied Linguistics (ARiEAL), Department of Linguistics and Languages, McMaster University, Hamilton, ON, Canada; ^6^Michael G. DeGroote School of Medicine, McMaster University, Hamilton, ON, Canada; ^7^Department of Psychiatry and Behavioural Neuroscience, McMaster University, Hamilton, ON, Canada; ^8^Mood Disorders Program, St. Joseph's Healthcare, Hamilton, ON, Canada; ^9^Department of Kinesiology, McMaster University, Hamilton, ON, Canada

**Keywords:** aerobic exercise, mindfulness, neurofeedback, intrinsic connectivity networks (ICN), default mode network (DMN), central executive network (CEN), salience network (SN), tri-network model

## Abstract

Lifestyle interventions such as exercise and mindfulness training have the potential to ameliorate mental health symptoms and restore dysregulated intrinsic connectivity network (ICN) dynamics, seen in many psychopathologies. Multiple lifestyle interventions, in combination, may interact synergistically for enhanced benefits. While the impacts of lifestyle interventions on subjective measures of mood are well-documented, their impacts on ICN dynamics are not well-established. In this study, we assessed the validity of EEG-derived measures of ICN dynamics as potential markers of mood disorders, by tracking ICN dynamics and mood symptoms through the course of a longitudinal exercise intervention. Specifically, we investigated the separate and combined effects of aerobic exercise and mindfulness-like neurofeedback training on task-linked ICN dynamics of the default mode network (DMN), central executive network (CEN), and salience network (SN). Participants were assigned pseudo-randomly into four experimental conditions—Control, Running, Neurofeedback, and Combined, performing the corresponding intervention for 16 sessions across 8 weeks. Intervention-linked changes in ICN dynamics were studied using EEG-based neuroimaging scans before and after the 8-week intervention, during which participants performed multiple blocks of autobiographical memory recall (AM) and working memory (WM) trials, designed to activate the DMN and CEN, respectively, and to activate the SN in conjunction with the task-appropriate network. The EEG-based features for classification of the three core networks had been identified in our prior research from simultaneously recorded EEG and fMRI during the same AM and WM tasks. We categorized participants as “responders” or “non-responders” based on whether the exercise intervention increased their aerobic capacity (VO2-max) (Running/Combined group), and/or neurofeedback increased the percentage time spent in the calm mindfulness state (Neurofeedback/Combined group). In responders, compared to each intervention alone, the combined exercise-neurofeedback intervention resulted in a more healthy CEN-SN synchrony pattern. Interestingly, non-responders to neurofeedback exhibited a maladaptive pattern of persistent, task-inappropriate DMN-SN synchrony which we speculate could be linked to depressive rumination. Furthermore, the CEN-SN synchrony at baseline predicted NFB response with up to 80% accuracy, demonstrating the potential utility of such network-based biomarkers in personalizing intervention plans.

## 1. Introduction

Mood disorders are becoming increasingly prevalent in the general population, and young adults are at a particularly elevated risk for depressive symptoms and suicidal thoughts (Chief Public Health Officer's Report on the State of Public Health in Canada, 2014). According to the WHO, suicide is the second global cause of death in those aged 15–29 (WHO, 2014). Moreover, youth in particular are much less likely to seek traditional treatments such as psychotherapy and pharmacotherapy for their mood disorders (Campo and Bridge, 2009), leaving them particularly vulnerable to the stressors in their lives. Making matters worse, traditional therapies are often expensive, stigmatizing—especially for youth, not universally accessible, and not always effective (Campo and Bridge, 2009; Wang et al., 2019). Therefore, accessible alternative intervention strategies are urgently needed to help youth at risk. Aerobic exercise and mindfulness training are two highly promising lifestyle interventions that overcome most or all of these barriers. Here, we investigate the separate and combined impact of exercise and a mindfulness-like neurofeedback protocol on mood and brain function in a cohort of young adults.

Exercise is well-established to ameliorate mood symptoms associated with depression (Cooney et al., 2013; Keating et al., 2018), as well as improve cognitive deficits including attention (Prakash et al., 2011; Chang et al., 2015), some forms of memory (Monti et al., 2012; Baym et al., 2014; Schwarb et al., 2017) and overall executive function (Guiney and Machado, 2013). It is also hypothesized to improve adult neurogenesis within the dentate gyrus (DG), associated with reduced memory interference (Déry et al., 2013), increased hippocampal volume and improved memory performance (Erickson et al., 2011). Such benefits may extend to the level of large-scale intrinsic connectivity networks (ICNs), with aerobic fitness explaining individual differences in functional connectivity within the central executive network (CEN), the default mode network (DMN), and dorsal/ventral attentional networks (DAN/VAN) (Talukdar et al., 2018). Even a single bout of moderate intensity aerobic exercise can enhance executive control-linked event related potentials (ERPs), indicating enhanced engagement of executive networks (Chang et al., 2015).

Mindfulness or meditation training is another lifestyle intervention found to be similarly effective in managing stress (Chiesa and Serretti, 2010), enhancing attention (Kozasa et al., 2012), enriching executive functioning (Gallant, 2016), and improving mood. Mindfulness training is also associated with neurbiological changes including increased grey matter in the cerebellum and posterior cingulate cortex (Holzel et al., 2011), increased telomerase activity associated with reduced oxidative stress (Epel et al., 2010; Jacobs et al., 2011), and increased synchronization between cardiac and brain activity (Gao et al., 2016) important for appropriate processing of emotional stimuli (Gray et al., 2012). Mindfulness based therapies lead to increased connectivity between the posterior cingulate cortex—a key node of the DMN, the dorsal anterior cingulate cortex (dACC)—a key node of the salience network (SN), and the dorsolateral pre-frontal cortex (dlPFC)—a key node of the CEN (Doll et al., 2015; King et al., 2016), and have shown promise in ameliorating PTSD symptoms (King et al., 2016). Such therapies can also modify the temporal dynamics of brain network connectivity, leading to a re-organization of EEG microstates post-therapy (Brechet et al., 2021). However, despite the benefits of mindfulness-based therapy, it traditionally requires a highly trained practitioner/clinician leading the mindfulness sessions. Much like traditional clinician-driven psychotherapies, this reduces the accessibility of such lifestyle-based interventions. One alternative is to use mindfulness-like therapies that can be self-administered, such as EEG-based neurofeedback (NFB) training, which have been found to be similarly impactful. A single NFB training session can upregulate connectivity of critical SN nodes, such as the dACC (Ros et al., 2013) and right insula (Kluetsch et al., 2014), in a manner similar to mindfulness training (Kilpatrick et al., 2011). Consequently, it has been found to be clinically useful in improving working memory, concentration, impulsivity, and dissociative symptoms in a wide range of psychopathologies, such as ADHD (Escolano et al., 2014; Thibault et al., 2016) and PTSD (Nicholson et al., 2016; Sitaram et al., 2016). While traditional clinical NFB training therapies rely on therapist-driven care, the advent of commercially available, low-cost EEG systems has led to the development of easy-to-use meditation-like NFB training devices such as the MUSE headband (InterAxon, Inc). When paired with a smartphone app on the user's phone, it can be used to administer EEG-based neurofeedback (NFB) training without the need for a trained clinician or practitioner. Hence, such a system could be a viable alternative to traditional mindfulness training protocols, while still providing similar cognitive and mood benefits.

While exercise and mindfulness interventions, each in their own right, have proven utility in mood disorders, there is also some evidence surrounding the synergistic benefits of combining these two interventions. For example, Mental and Physical (MAP) training, a combined meditation and aerobic exercise intervention, improves symptoms of depression and anxiety, hypothetically through improved hippocampal neurogenesis (Shors et al., 2014). This hypothesis is based on observations in rodents that although exercise upregulates hippocampal neurogenesis, a sizeable proportion of new neurons are lost to programmed apoptosis (Curlik and Shors, 2013); in contrast, exercise paired with cognitive training increases both neurogenesis and neural survival (DiFeo and Shors, 2017). Given the crucial role of hippocampal circuits in large-scale brain network dynamics (Shaw et al., 2021a), a combined aerobic exercise and mindfulness training protocol has the potential to ameliorate dysfunctional network dynamics in individuals with a range of psychiatric disorders such as PTSD and bipolar disorder. In fact, MAP training was found to be effective in reducing post-traumatic cognitions and ruminative thoughts in a population of PTSD patients (Shors et al., 2018).

Studies using MAP training have shown that combined mental training and physical activity can act synergistically to reduce ruminative thoughts and post-traumatic cognitions, relative to each intervention alone (Shors et al., 2018), however, the effect of exercise and NFB training on task-related brain network dynamics is not well-understood. While some studies have investigated exercise or NFB-linked brain network changes (Kluetsch et al., 2014; Talukdar et al., 2018; Brechet et al., 2021), none have studied the combined effect of both interventions. Furthermore, most of these studies probe the resting-state dynamics of large-scale brain networks and do not investigate the intervention-linked changes in network dynamics under task loads. Moreover, group mindfulness classes, though effective, are not readily available or manageable by everyone. There is a need to investigate more accessible mindfulness alternatives such as mindfulness-like NFB training.

The current study was designed to address these knowledge gaps by investigating the separate and combined effects of aerobic exercise and mindfulness-like NFB training on task-related brain network dynamics in a cohort of healthy young adults.

We predicted that aerobic exercise and mindfulness-like NFB training would each lead to improvements in the participants' task-appropriate network synchrony, in addition to improving their aerobic capacity and their NFB scores, respectively (hypothesis 1). We further hypothesized that the two interventions combined would produce a synergistic effect and lead to even greater enhancement of task-appropriate synchrony, relative to that seen in the single intervention groups (hypothesis 2).

## 2. Methods

### 2.1. Study Design

**Figure 1:**
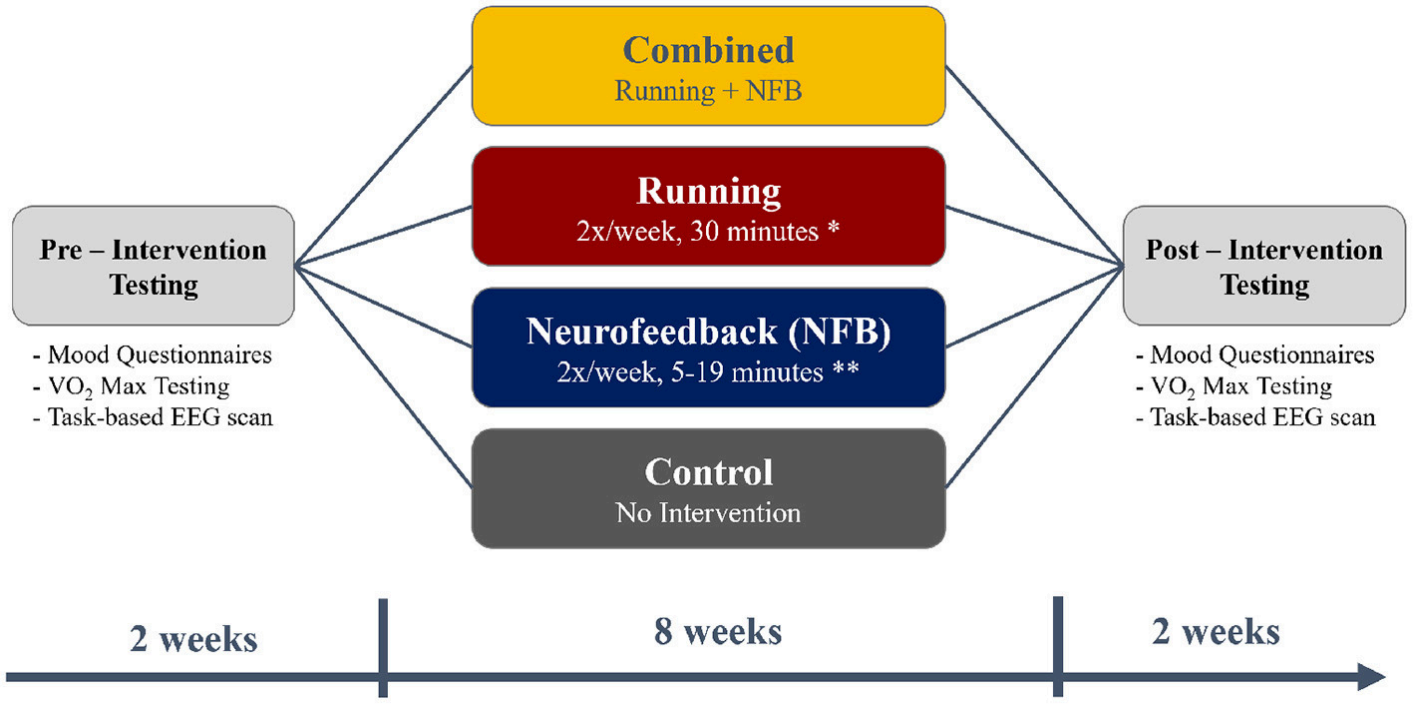
A flowchart depicting the timing of the pre-intervention testing (2 weeks), the intervention (8 weeks), and post-intervention testing (2 weeks). The timings were chosen to fit within the first 3 months of the 4-month long university term.

A total of 140 young adults were recruited from McMaster University for the current study, after excluding participants who answered “Yes” to any of the questions in the Get Active Questionnaire (GAQ) that would contraindicate participation in an exercise program. Participants were also excluded if they regularly exercised more than 1 h a week. A total of 15 participants were excluded based on these exclusion criteria. After recruitment was complete, each participant was pseudo-randomly assigned to one of the four experimental groups - Control, Running, NFB, and Combined (Running + NFB), as shown in Figure 1 and further detailed below.

1. Those in the **Running** group participated in an 8-week aerobic exercise protocol consisting of two sessions of 24 min each week. Inspired by Galloway (2016)'s Run-Walk-Run method, each 24 min session was further divided into eight intervals of 3 min each, consisting of running and walking. The difficulty of each session was increased over time by increasing the proportion of time each week participants spent running in each 3 min interval (see Table 1).
2. Those within the **Neurofeedback (NFB)** group performed mindfulness-like NFB training with the Muse headband along with its companion Calm app, further described in Section 2.1. This NFB training was performed two times a week, for an 8-week period. Similar to the exercise protocol, the difficulty of the NFB sessions was increased over time by increasing the session lengths as the participants progressed through the weeks, from 5 min sessions in the first week to 19 min sessions in the eighth week (see Table 1).
3. Finally, participants in the **Combined** group (Running + NFB) performed both the NFB training and the aerobic exercise protocol at each session. The NFB training was performed before the aerobic exercise at each session.
4. Participants assigned to the **Control** group did not participate in any of the training protocols, and were simply asked to carry on with their current level of activity.

**Table 1:** The running and NFB protocols used in this study.

**Week**	**Running protocol**		**Neurofeedback protocol**
	**Run interval**	**Walk interval**	**NFB length**
1	1:00	2:00	5:00
2	1:15	1:45	7:00
3	1:30	1:30	9:00
4	1:45	1:15	11:00
5	2:00	1:00	13:00
6	2:15	0:45	15:00
7	2:30	0:30	17:00
8	2:45	0:15	19:00

*Each running session consisted of eight 3 min intervals, which were split into varying amounts of running and walking, as shown in the table. The difficulty of the running sessions increased every week by increasing the proportion of time spent running in each 3 min interval. In contrast, the NFB difficulty was increased by increasing the length of time the participants spent performing mindulness-like NFB*.

Before and after the 8-week intervention, each participant also completed mood questionnaires, an assessment of aerobic capacity (VO2-max assessment) and a task-based neuroimaging scan, as illustrated in Figure 1.

#### 2.1.1. NFB Intervention Equipment

The NFB training sessions used the MUSE headband (InterAxon, Inc) along with its companion CALM app to administer the mindfulness-like NFB training protocol. This combination (MUSE headband + CALM app) has been previously validated for administering a mindfulness-like NFB training protocol in a randomized control trial (Bhayee et al., 2016). Furthermore, the efficacy and reliability of the MUSE headband in adequately recording EEG-based features, such as event-related potentials (ERPs), has been tested and verified by multiple studies (Krigolson et al., 2017, 2021).

#### 2.1.2. VO2-Max Assessment

Maximal oxygen uptake, i.e., VO2-max, is a strong indicator of cardiorespiratory fitness and was assessed using a graded aerobic fitness test (VO2-max) on a digital cycle ergometer (Lode Groningen, The Netherlands). The test protocol consisted of a 30 s warm-up period at 50 W of resistance, followed by an increase in resistance of 1 W every 2 s, until the participant reached exhaustion. Verbal encouragement was provided to the participants and they were instructed to maintain a speed above 80 rpm. The test was terminated when the participants voluntarily noted their exhaustion. Throughout the test, heart rate (HR) was monitored using a Polar heart rate monitor, and expired gases were monitored *via* a breathing tube on the participants' mouths that led to a metabolic cart (MEDISOFT ExpAir Software VO2 System; Dinant, Belgium). The maximum oxygen uptake during this test was recorded as the participants' VO2-max score. This protocol is routinely used to assess participants' VO2-max, as described by Gillen et al. (2014) and Lucibello et al. (2019).

#### 2.1.3. Neuroimaging Scan

A task-based neuroimaging scan was performed before and after the interventions to estimate the participants' level of task-appropriate network synchrony. High resolution EEG data were recorded from a 128-channel BioSemi ActiveTwo EEG system while participants performed multiple blocks of an autobiographical memory recall (AM) and a 2-back working memory task (WM), randomly interleaved. The participants were shown personalized words from a neutral or positive memory during the AM blocks and were instructed to think about their memory in detail. These personalized words were derived from salient details of 10 neutral or positive memories that were collected from the participant before the start of the neuroimaging scan. During the WM blocks, the participants were shown words from a generic word bank and asked to remember the word they saw two words ago. Each task block lasted 30 s in total. This task paradigm is identical to that used in Shaw et al. (2021a) and is designed to activate the three intrinsically connected networks (ICNs) of the tri-network model (Menon, 2011), namely, the default mode network (DMN), the central executive network (CEN), and the salience network (SN).

The recall of episodic memories during the AM blocks was expected to recruit the DMN and its substructures (Addis et al., 2004; Svoboda et al., 2006; Daselaar et al., 2008; Andrews-Hanna et al., 2014), while the use of executive processing and working memory resources during the WM blocks were expected to recruit the CEN and its substructures (Smith and Jonides, 1997; Collette and Van der Linden, 2002; Owen et al., 2005; Yaple et al., 2019). Moreover, Shaw et al. (2021a) showed that an identical task switching paradigm in an fMRI study, led to increased DMN-SN co-activation (coupling) during AM task blocks and increased CEN-SN co-activation (coupling) during WM task blocks, suggesting that the SN co-activates with the task appropriate network.

#### 2.1.4. Recruitment Details

All research protocols used in the study were reviewed and approved by the McMaster University Research Ethics Board (MREB). Of the 140 recruited participants, 69 participants aged 20.8 ± 3.7 years (10 males) completed the full intervention protocol and pre/post-intervention testing, with an attrition rate of 50%. This included 15 participants in the Control group, 20 participants in the Running group, 14 participants in the NFB group, and 20 participants in the Combined group.

### 2.2. EEG-Based Network Analysis

To study intervention-linked changes to large-scale brain networks, the task-based EEG data from the pre-intervention and post-intervention testing sessions were processed using a previously established analysis pipeline designed to classify ICN activation using EEG data alone, as described in Shaw et al. (2021b). This analysis pipeline included pre-processing of the EEG data, extraction of features relevant to the three ICNs of interest: the DMN, CEN, and SN, and finally prediction of ICN activation probability based on the generalized model provided in Shaw et al. (2021b). A list of all abbreviations used in the manuscript is provided in Table 2. These steps are summarized below (refer to Shaw et al., 2021b for further details).

**Table 2:** A list of abbreviations used in this manuscript.

**Abbreviation**	**Terminology**	**Abbreviation**	**Terminology**
EEG	Electroencephalography	DAN/VAN	Dorsal/Ventral attention network
ICN	Intrinsic connectivity network	dACC	Dorsal anterior cingulate cortex
AM	Autobiographical memory	dlPFC	Dorsolateral prefrontal cortex
WM	Working memory	MAP training	Mental and physical training
DMN	Default mode network	NFB	Neurofeedback
CEN	Central executive network	ICA	Independent component analysis
SN	Salience network	PCE	Pairwise cross-entropy

#### 2.2.1. EEG Pre-processing

EEG data were first temporally filtered into six different frequency bands—full (1–50 Hz), delta (1–4 Hz), theta (4–8 Hz), alpha (8–13 Hz), beta (13–30 Hz), and gamma (30–50 Hz), using a zero phase lag 12-order Butterworth IIR filter. This was followed by detection and removal of bad channels based on their spectral characteristics. An ICA decomposition of the EEG data was then performed using the FAST-ICA algorithm implemented within EEGLAB (Delorme and Makeig, 2004), followed by removal of artifactual ICA components (ocular artifacts, eye blinks and muscle artifacts) using the automated ICLabel tool. Finally, the EEG data were average referenced, followed by spherical interpolation of the bad EEG channels identified earlier (interpolation done only if the number of bad channels was <5% of the total number of channels). The EEG data were finally epoched, creating windows of length 5 s with no overlap. The epochs were aligned with the onset of each 30 s task block.

On average, 2.5 ± 1.4 electrodes were identified as bad channels and removed at the beginning of pre-processing, while ~16.3 ± 2.7 ICA components were identified as artifactual during the ICA-based artifact removal step. More importantly, the mean number of bad channels and artifactual ICA components did not differ between the participant groups.

#### 2.2.2. Feature Extraction

The pre-processed EEG data were used to compute the set of 5,000 optimal connectivity features identified in Shaw et al. (2021b) that uniquely identify activation of the CEN, DMN, and SN. These included coherence, phase lag index, directed phase lag index, phase amplitude coupling, and cross-frequency coupling (synchronization index) across the six previously computed frequency bands. Shaw et al. (2021b) derived these features as representative signatures of the CEN, DMN, and SN activation using a simultaneously recorded EEG-fMRI dataset collected from participants performing an AM-WM switching task paradigm, identical to that used in this study.

#### 2.2.3. ICN Activity Prediction

The computed features were used with the generalized model trained in Shaw et al. (2021b) (available for download at https://github.com/saurabhshaw/EEGnet) to predict the probability of each of the three ICN's activation at each time window. This provided the probability of DMN (*P*_*dmn*_), CEN (*P*_*cen*_), and SN (*P*_*sn*_) activation over 5 s windows of EEG data. The pairwise cross-entropy (PCE) between the probability distributions of each network pair was computed to estimate their similarity/synchronization over the course of the WM and AM trials. For example, Equation (1) shows the process of estimating the PCE between the DMN and SN networks over *M* trials of either the WM or AM task. According to this definition, a lower *PCE*_*dmn*−*sn*_ corresponds to higher similarity between the probability distributions of DMN activation and SN activation across multiple trials of either WM or AM task, which can be interpreted as higher DMN-SN synchronization over the *M* trials.

(1) $$\begin{array}{r} PCE_{dmn-sn}=-\overset{M}{\underset{i=1}{\sum }}P_{dmn}\times log\left(P_{sn}\right) \end{array}$$

This was performed across the entire length of the WM and AM trials, followed by separately estimating the PCE across the first half and second half of each of the WM and AM trials. The analysis was split between the first half and second half of the AM and WM tasks as prior work showed that task demands and network activation were different during the first and second halves of these tasks (Shaw et al., 2021a). As an example, when a participant was performing a 30 s AM block, they tended to activate the DMN more strongly during the first half of the trial, and then transition to CEN activation during the latter half of the trial, presumably because they were recruiting the DMN initially to retrieve the memory, but then relying on the CEN to maintain the retrieved memory in working memory.

### 2.3. Determination of Responder/Non-responder Status

It is well-established in the literature that substantial variance exists in the response to NFB training protocols and exercise protocols. A recent meta-analysis revealed that 16–57% of the participants were unable to learn to control their brain state to match the NFB target state (Alkoby et al., 2018; Weber et al., 2020), commonly referred to as the “inefficiency” problem. Similarly, for a variety of reasons including genetic factors and motivation (Ross et al., 2019), responses to aerobic exercise protocols are highly variable. Hence, a widespread practice is to divide participants into responders and non-responders to the intervention (Déry et al., 2013; Pickering and Kiely, 2019; Weatherwax et al., 2019; Metcalfe and Vollaard, 2021).

Based on the above evidence, to account for this variance in the participants' ability to respond to the intervention, we labeled each participant as either a responder or non-responder based on the change in their intervention-relevant outcome variable, as described below.

1. **NFB Group:** Two different outcome measures were used to assess the efficacy of the NFB intervention due to its bi-factorial nature, merging mindfulness-like aspects with NFB training. The first dependent variable was the slope of the percentage of time spent in the “calm zone” over the course of the protocol. This provided an estimate of the participants' meditative ability. The second dependent variable used was the slope of the number of points awarded by MUSE vs time, within each NFB session. Although linked to the time spent in the calm zone, this measure was more directly linked to the NFB performance of the participants.
  Participants were classified as responders/non-responders based on the percentage of time they spent in the “calm state” during each session, and the MUSE score achieved during each session. For each of these measures, we calculated the slope of the line of best-fit over the course of the 8 week-long intervention. Participants were considered non-responders if neither of these measures improved over the course of the intervention, i.e., no change (zero slope) or decreased (negative slope). Otherwise, if either or both measures improved (positive slope), they were considered to be a responder.
2. **Running Group:** Participants that had the same or lower VO2-Max level at the post-intervention timepoint, compared to that at the pre-intervention timepoint, were deemed non-responders. Participants were considered responders if their VO2-Max level at the post-intervention timepoint was higher than that at the pre-intervention timepoint (positive change in VO2-Max).
3. **Combined Group:** Participants were considered responders to the combined intervention if they satisfied the responder requirements for both the NFB intervention and the aerobic exercise intervention, as described above. Participants that did not satisfy both responder requirements were considered non-responders.

### 2.4. Analysis of Intervention Effectiveness

As a manipulation check, we first assessed the effectiveness of the aerobic exercise intervention on aerobic fitness by conducting a one-way ANOVA with participant group (four levels) as the independent factor, and the change in each participants' VO2-max level from baseline (Pre-timepoint to Post-timepoint) as the dependent factor. This was followed up with Bonferroni-corrected independent samples *t*-tests to understand the groups differences in VO2-max levels. The Bonferroni-corrected threshold for significance was 0.0125 (0.05/4).

Similarly, we assessed the effectiveness of the mindfulness-like NFB intervention by conducting a multivariate one-way ANOVA with participant group (four levels) as the independent factor, and the two measures of the participants' ability to stay in the target “calm” brain state, as the dependent variables.

Both of these analyses were also corrected for multiple comparisons using false discovery rate (FDR) correction.

### 2.5. Analysis of Network Changes

After the effectiveness of the interventions were verified, the impacts of the intervention on PCE, our primary outcome measure of network synchrony, were assessed.

To do this, we first performed a multivariate three-way ANOVA with all four PCE measures of interest as the outcome variables (DMN-SN during WM, DMN-SN during AM, CEN-SN during WM, and CEN-SN during AM), and Group, Session, and Responder status as the three factors. The significant findings from this all-encompassing ANOVA analysis motivated follow-up analyses to understand the nature of the differences observed.

The follow-up analyses included two two-way repeated measures ANOVAs (with Group as the between-group factor and Session as the within-group factor) with each of the two PCE measures found to be significant in the previous analysis as the outcome variables. This was performed for the responders and non-responders separately, using bootstrapping with 1,000 samples to minimize the effect of outliers. These analyses were corrected for multiple comparisons using false discovery rate (FDR) correction.

The above ANOVAs were followed up with *post-hoc* pairwise one-tailed independent *t*-tests between each pair of groups to identify the experimental group driving the observed results, corrected for multiple comparisons using false discovery rate (FDR) correction.

### 2.6. Machine Learning Analysis

To investigate whether the above measure of ICN cross-network synchronization was predictive of participants' neurofeedback performance (responder status), the *PCE*_*dmn*−*sn*_ and *PCE*_*cen*−*sn*_ at the pre-intervention timepoint were used as predictor variables to classify NFB response (as defined in Section 2.3). This was performed using a linear support vector machine (SVM) with 10-fold cross-validation.

## 3. Results

### 3.1. Manipulation Check: Intervention Effectiveness

**Figure 2:**
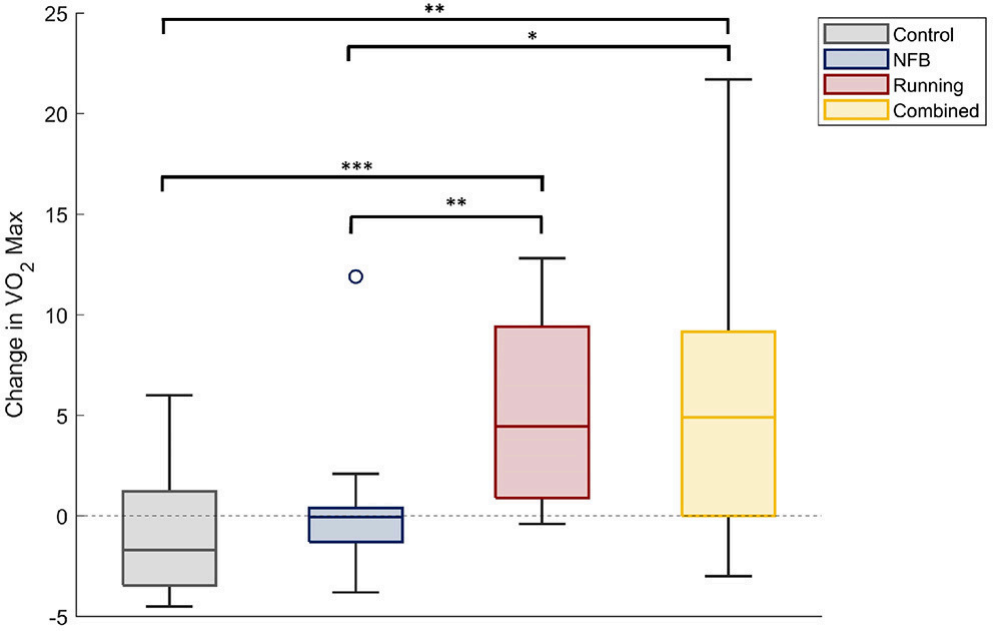
The change in VO2-max scores of participants within each experimental group. The Running and Combined groups show a significant increase in VO2Max compared to the Control and NFB groups. Significance of *post-hoc* pairwise independent samples *t*-tests are represented by *p < 0.05, **p < 0.01, and ***p < 0.001.

A one-way ANOVA with participant group (four levels) as the independent factor, and the change in each participants' VO2-max level from baseline (Pre-timepoint) as the dependent measure, revealed a significant effect of group [*F*_(3,33)_ = 3.53, p-FDR = 0.025, η^2^ = 0.243]. Participants in the Running and the Combined groups showed a large increase in their VO2-max levels, compared to those in the Control and NFB groups (shown in Figure 2), as revealed by *post-hoc* independent samples *t*-tests thresholded at a Bonferroni-corrected alpha level of 0.0125 (0.05/4); specifically, there were significant differences between the Running and Control groups [*t*_(36)_ = 4.548, *p* < 0.001, dz = 0.738], Combined and Control groups [*t*_(32)_ = 3.401, *p* = 0.002, dz = 0.567], Running and NFB groups [*t*_(38)_ = 3.088, *p* = 0.004, dz = 0.690], and a marginally non-significant difference between the Combined and NFB groups [*t*_(34)_ = 2.574, *p* = 0.015, dz = 0.429]. The exercise protocol employed in this study was thereby effective in increasing the aerobic capacity of the participants.

**Figure 3:**
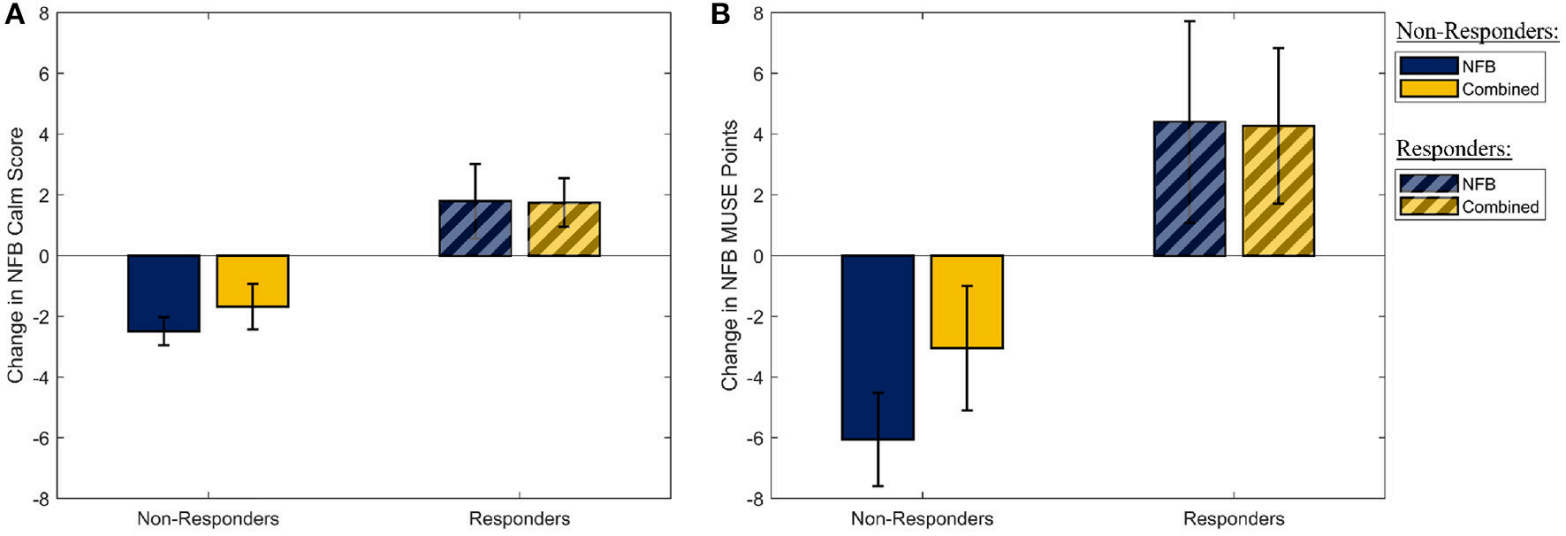
The distribution of the two metrics used to determine NFB “Responders” and “Non-Responders.” **(A)** The mean slope of the amount of time participants spent in the “Calm” state during their mindfulness-like NFB training sessions. **(B)** The mean change in the MUSE points during each mindufulness-like NFB training sessions. Error bars shown are ± 1 SE.

Similarly, assessing the effectiveness of the mindfulness-like NFB intervention, a multivariate one-way ANOVA with participant group (four levels) as the independent factor, and the measures of the participants' ability to stay in the target “calm” brain state as the dependent measures, revealed no statistically significant effect of the NFB protocol on either of the two outcome measures [*F*_(1,30)_ = 0.31, p-FDR = 0.737, η^2^ = 0.021]. However, as it is well-established that a sizeable proportion of participants are unable to learn to control their brain state through EEG-NFB (see Section 2.3), these measures were used to split the participants into responders and non-responders on the basis of their success in increasing their NFB performance metric over the 8-week period. The two NFB performance metrics (change in NFB “calm” score, and change in MUSE points) are shown in Figure 3 for the responders and non-responders separately. A total of 16 responders were identified across the NFB and Combined groups (11 responders in the NFB group, 5 responders in the Combined group).

### 3.2. Network Dynamics

To study the impact of the interventions on task-dependent network dynamics, the co-activation of the CEN and DMN with the SN were studied by assessing the synchronous activation of the SN with each task-appropriate network [i.e., the DMN for AM trials, CEN for WM trials (Shaw et al., 2021a)] vs the task-inappropriate network (i.e., CEN for AM trials, DMN for WM trials). To assess synchrony between networks, we measured the pairwise cross-entropy (PCE) between the task-linked probability distribution of CEN or DMN activation and that of SN activation during corresponding task intervals. The PCE between each pair of networks was expected to be lower for higher synchronization/co-activation of the networks with the SN, with a PCE of zero indicating maximum synchrony between networks. The interventions were expected to shift participants' network dynamics toward a more healthy pattern of network co-activation, in which the SN co-activates with the task-appropriate network a greater proportion of the time. Hence, the interventions were expected to decrease the PCE between DMN and SN during the AM trials, and between the CEN and SN during the WM trials, indicative of increased synchrony between the SN and the task-appropriate network. Conversely, the interventions were expected to increase the PCE between the DMN and SN during the WM trials, and between the CEN and SN during the AM trials, indicative of less synchrony between the SN and the task-inappropriate network.

First, a multivariate three-way ANOVA was performed with the four above mentioned PCE measures as outcome variables, and Group, Session and Responder as factors. This analysis found a significant Group x Session x Responder interaction for the PCE between DMN and SN [*F*_(2,46)_ = 4.139, *p* = 0.022, η^2^ = 0.016], and CEN and SN [*F*_(2,46)_ = 4.876, *p* = 0.012, η^2^ = 0.024] during the WM trials. On the other hand, no such interaction effect was observed for the PCE between DMN and SN [*F*_(2,46)_ = 2.741, *p* = 0.075, η^2^ = 0.008], and CEN and SN [*F*_(2,46)_ = 1.124, *p* = 0.329, η^2^ = 0.002] during the AM trials. On the basis of this all-encompassing ANOVA, the PCE measures during AM trials were excluded from subsequent analyses.

To further investigate these findings, two-way repeated measures ANOVAs (with Group as the between-group factor and Session as the within-group factor) were performed with each of the two PCE measures found to be significant in the previous analysis as the outcome variable. This was performed for the responders and non-responders separately, using bootstrapping with 1,000 samples to account for outliers and small sample sizes. These findings are discussed below.

#### 3.2.1. NFB Non-responders Show Increased Task-Inappropriate DMN Synchronization With SN

**Figure 4:**
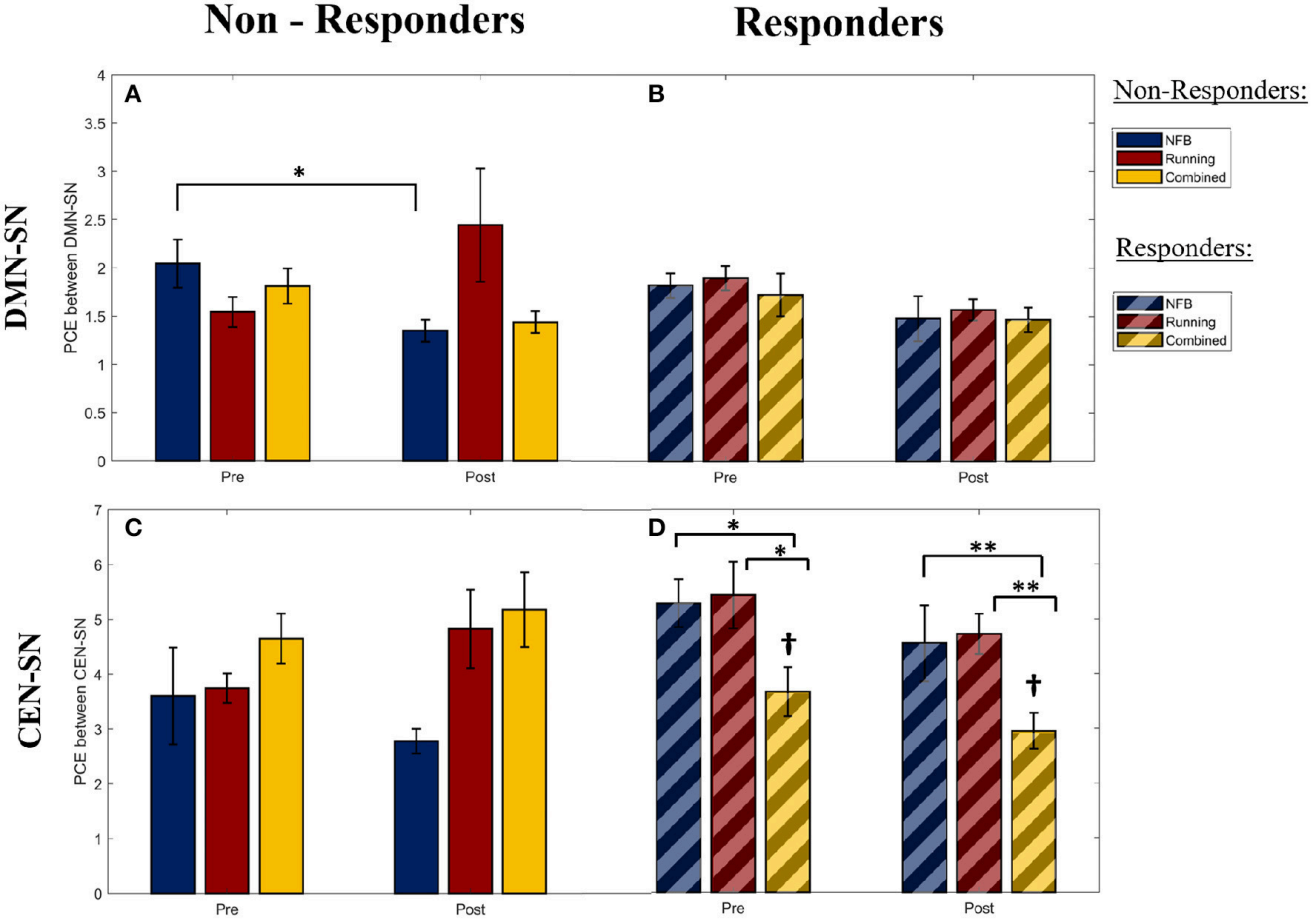
The pairwise cross-entropy (PCE) between the DMN/CEN and SN networks during WM trials (a lower PCE value indicates higher DMN/CEN-SN synchrony). PCE is calculated for the DMN-SN pair within the Non-Responders **(A)** and Responders **(B)**, for the CEN-SN pair within the Non-Responders **(C)**, and Responders **(D)**. Significance of *post-hoc* pairwise independent samples *t*-tests are represented by *p < 0.05 and **p < 0.01. ^†^Represents a significant difference (*p* < 0.05) with respect to Non-Responders within the Combined group at the corresponding timepoints. Error bars shown are ± 1 SE.

Within non-responders, a significant main effect of Session was observed for the PCE between DMN and SN during the WM trials [*F*_(1,25)_ = 4.41, p-FDR = 0.046, η^2^ = 0.320]. The DMN-SN PCE was found to be significantly lower (i.e., higher synchronization in the task-inappropriate network) [*post-hoc* 1-tailed *t*-test *t*_(2)_ = −4.661, p-FDR = 0.045, dz = 2.69] in the non-responders within the NFB group at the post-intervention session, compared to the non-responders in the Combined group, as seen in Figure 4A. This was not observed in the responders within the corresponding groups, as seen in Figure 4B. This implies that the DMN was more (inappropriately) synchronized with the SN during the WM trials in the non-responders within the NFB group, indicating that NFB non-responders could have shifted their task-linked network dynamics toward a more dysregulated state.

Furthermore, this pattern of higher DMN-SN synchronization (lower DMN-SN PCE) at the post-intervention timepoint in non-responders of the NFB was also observed in both halves of the WM trials [First half: *F*_(1,25)_ = 3.398, p-FDR = 0.041; Second half: *F*_(1,25)_ = 5.968, p-FDR = 0.018]. This is in contrast to the healthy pattern of DMN deactivation observed during the second half of the WM trials, seen in previous research studies (Shaw et al., 2021a). This finding further suggests that the non-responders within the NFB group might have succumbed to the increasingly stressful demands throughout the school semester, and therefore had increasingly greater trouble deactivating the DMN according to changing task demands.

#### 3.2.2. Addition of Aerobic Exercise to NFB Training Rescues Task-Inappropriate DMN-SN Synchronization Within NFB Non-responders

Interestingly, the pattern of lower DMN-SN PCE (higher synchronization in the task-inappropriate network) observed within the non-responders of the NFB group was not observed in the non-responders of the Combined group (Figure 4A). This suggests that there could be a protective role of aerobic exercise in minimizing maladaptive network dynamics. However, the interaction effect of Group x Session for the non-responders in the NFB and Combined groups in this study showed a trend effect and did not reach statistical significance after correcting for multiple comparisons [*F*_(1,25)_ = 1.37, p-FDR = 0.253, η^2^ = 0.284], potentially due to limited sample size. Hence, this prediction needs to be further investigated in future studies with a larger sample size.

#### 3.2.3. CEN More Synchronized With SN During WM Trials in Responders Within the Combined Group

Within responders of all intervention groups, a significant main effect of Group was observed for the PCE between CEN and SN during the WM trials [*F*_(2,38)_ = 4.108, p-FDR = 0.024, η^2^ = 0.468]. As seen in Figure 4D, the responders within the combined group show a lower CEN-SN PCE (higher synchronization of the task-appropriate network) compared to responders in the other groups, and non-responders within the combined group (Figure 4C). Given that there was no main or interaction effect of Session (Pre/Post) on the CEN-SN PCE, this difference was present at the start of the intervention and was not a result of the intervention. This implies that the participants with higher CEN-SN synchronization during executive tasks at baseline, and that participated in aerobic exercise, were able to perform better on the mindfulness-like NFB task of staying within the “calm state”. Hence aerobic exercise modulated the impact of CEN-SN synchrony on NFB training.

When this pattern of CEN-SN PCE was examined across the two halves of the WM trials, the CEN-SN PCE did not show a main effect of Group during the first half [*F*_(2,38)_ = 2.819, p-FDR = 0.067], while it did show a main effect of Group during the second half of the WM trials [*F*_(2,38)_ = 3.556, p-FDR = 0.035]. Therefore, the higher CEN-SN synchronization observed within the responders of the combined group during the WM trials is due to an increase in CEN-SN synchronization during the second half of the WM trials, similar to the healthy pattern of network dynamics observed during WM (Shaw et al., 2021a). This implies that the network dynamics within this group of participants better follows task demands, and could be the reason they were successful at the NFB task.

#### 3.2.4. CEN-SN Synchronization at Baseline Predicts NFB Response

To further test the link between CEN-SN synchronization and NFB response suggested in Section 3.2.3, the PCE between CEN and SN at the pre-intervention time point was used as the predictor variable to classify NFB response (calm state). The resultant 10-fold cross-validated classification accuracy using a linear support vector machine (SVM) was 77.5 ± 12.4%. This implies that a participant's response to future NFB training could be predicted solely based on their current degree of CEN-SN synchronization during a working memory task.

Interestingly, performing this classification task within the NFB group and the combined group separately did not show (*p* = 0.72) better classification accuracy for the combined group (80.0 ± 25.8%), compared to the NFB only group (75.0 ± 35.3%).

#### 3.2.5. Increased VO2-Max Scores Associated With Improved Task-Appropriate Network Synchrony During WM Trials

**Figure 5:**
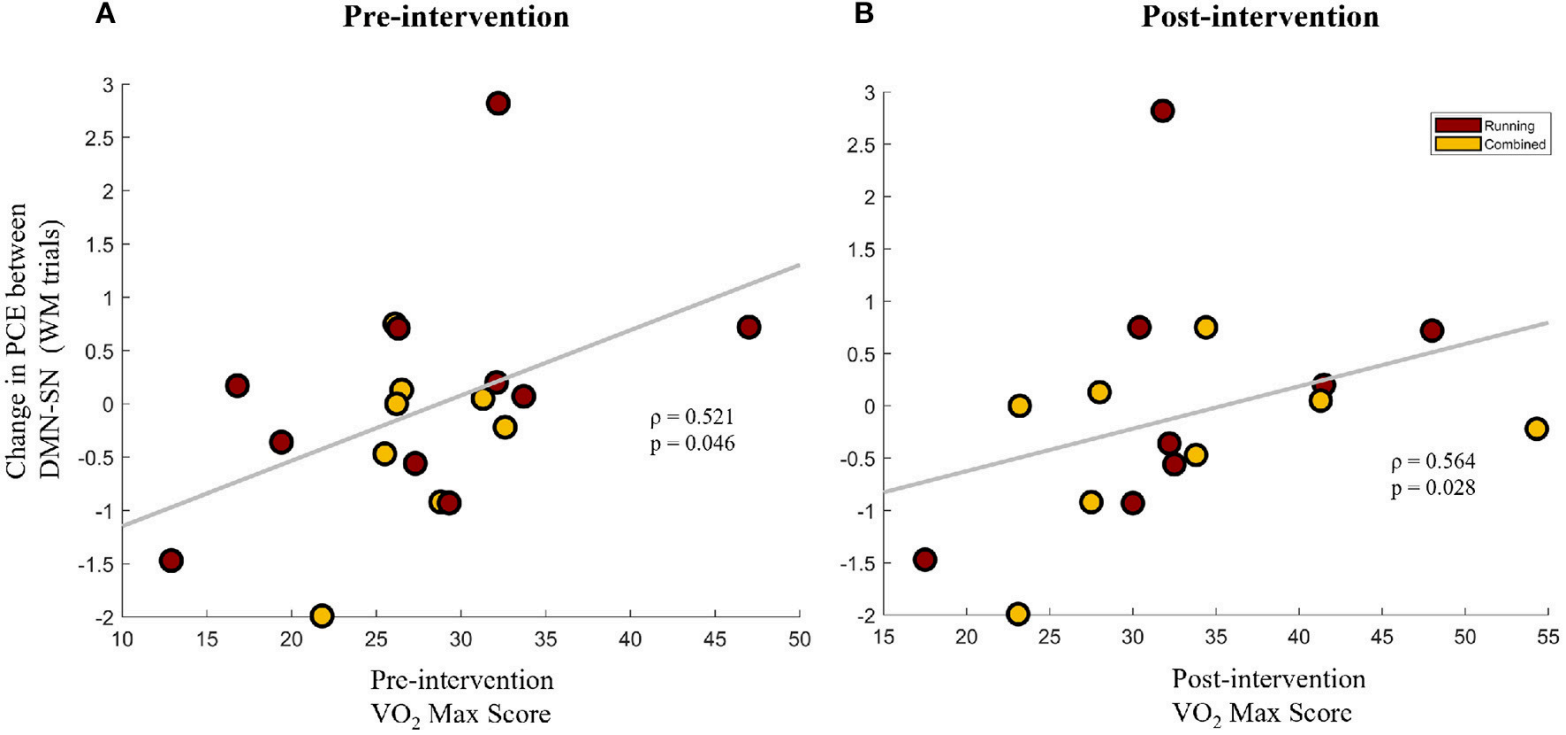
The change in pairwise cross-entropy (PCE) between the DMN and SN networks during WM trials (a lower PCE value indicates higher DMN-SN synchronization) for the participants that participated in the aerobic exercise protocol (Running group + Combined group). Change in PCE is shown against the VO2-Max scores of the participants at the pre-intervention timepoint **(A)** and the post-intervention timepoint **(B)**.

To further understand the association between improvements in aerobic fitness and brain network synchrony, the change in network synchrony (Post-Pre) was correlated with VO2-Max scores of the participants that participated in the aerobic exercise protocol (Running group + Combined group). This was done separately for the VO2-Max scores at the pre-intervention and post-intervention timepoints. Interestingly, the change in the PCE between the DMN and SN during the WM trials was found to be positively correlated with VO2-Max scores at baseline (Spearman's ρ = 0.521, *p* = 0.046) and at the post-intervention timepoint (Spearman's ρ = 0.564, *p* = 0.028), as shown in Figures 5A,B, respectively. Given the inverse relationship between PCE and network synchrony, these results imply a negative association between VO2-Max scores and the task-inappropriate DMN-SN synchrony during WM trials. Combined with the findings of Section 3.2.2, these results indicate that greater aerobic fitness is associated with greater improvements in task-appropriate network synchrony. Furthermore, since pre-intervention VO2-Max scores are positively associated with future improvements in task-appropriate synchrony, baseline fitness could be priming a healthy change in network synchrony.

## 4. Discussion

The promising mood and neurobiological benefits of lifestyle interventions such as aerobic exercise and mindfulness training (Shors et al., 2014, 2018) could potentially make them an effective prophylactic measure to prevent mental health deterioration in vulnerable and at-risk populations. However, their widespread adoption is limited due to the requirement for trained meditation and exercise coaches, making them prohibitively expensive for at-risk populations with limited resources such as undergraduate university students. Furthermore, despite evidence for the benefits of combining multiple lifestyle interventions (Shors et al., 2014), the impact of combining such lifestyle interventions on task-based dynamics of large-scale brain networks is poorly understood. This study is a first step toward understanding task-related changes in large-scale brain networks as a result of either a running-based aerobic exercise protocol, or a self administered mindfulness-like NFB protocol, or both in combination. This well-controlled approach of studying each lifestyle intervention separately, and then in combination, allowed us to identify intervention-linked changes in large-scale brain network dynamics that are unique to one intervention, and further showed the added benefit of combining these interventions. Finally, the self-administered nature of the NFB protocol increases the accessibility of such an intervention for populations with limited resources, addressing one of the major barriers for the adoption of such interventions.

Interestingly, participants “resistant” to NFB training (non-responders within the NFB group) were also more likely to engage in task-inappropriate DMN-SN synchronization during the WM trials. This abnormal DMN recruitment during the WM trials implies that non-responders within the mindfulness-like NFB training group adopted the maladaptive strategy of persistently activating the DMN, even during executive tasks. Further evidence of DMN-SN synchronization during the second half of the WM trials, during which the DMN is expected to be deactivated (Shaw et al., 2021a), suggests that the NFB non-responders might have difficulty suppressing the DMN when needed. Such maladaptive strategies can be particularly problematic since they can result in poor cognitive performance (Anticevic et al., 2012), higher mind-wandering (Xiao et al., 2019), and higher levels of rumination, as seen in psychopathologies such as MDD (Goncalves et al., 2017) and OCD (Hamilton et al., 2015).

However, the results further indicate that such maladaptive strategies linked to NFB non-response can be mitigated by engaging in aerobic exercise in addition to mindfulness-like NFB training. Results of Section 3.2.5 suggest that higher aerobic fitness might be priming the suppression of task-inappropriate DMN-SN synchrony. Hence, the increased aerobic fitness of participants in the Combined group (Figure 2) could be preventing the task-inappropriate DMN-SN synchrony observed in those “resistant” to the NFB training protocol. While the exact mechanism of such a synergistic action is yet unknown, the aerobic exercise might act through hippocampal circuits which are known to feed into posterior DMN nodes, such as the posterior cingulate cortex (PCC). These nodes are the primary foci of DMN-linked changes after a short mindfulness training protocol (Xiao et al., 2019), and could be modulated by the hippocampal changes brought about through aerobic exercise. Another mechanism of action could be improvements in CEN dynamics and executive processing often linked with aerobic exercise (Chang et al., 2015). CEN activation can directly result in deactivating the DMN (Chen et al., 2013), resulting in the suppression of any maladaptive mind-wandering or ruminative behaviors.

In fact, the CEN was found to be more synchronized with SN during the task-appropriate WM trials within responders of the Combined group, compared to non-responders within the Combined group. This difference was even seen at the pre-intervention timepoint and could predict whether a participant would be able to successfully perform the NFB training with up to 80% accuracy.

Predicting NFB response is a particularly important, albeit challenging task. If possible, such predictions could enable personalized intervention plans for each participant, maximizing the impact of such protocols. While recent literature-wide surveys find that current predictors are incapable of predicting NFB response in healthy and clinical populations (Weber et al., 2020), this study shows that NFB response can be predicted with the participants' task-based CEN-SN synchrony. These results highlight the importance of novel EEG-based biomarkers (Shaw et al., 2021b), that have great clinical potential in informing personalized therapy plans and improving overall treatment efficacy. The novel analysis pipeline used in this study also allowed the use of the cheaper and more accessible EEG modality to assess ICN synchrony, which typically involves more expensive functional imaging modalities such as fMRI. This greatly increases the clinical accessibility of such biomarkers, reducing the barriers to adopt such biomarker-based predictive strategies to treatment planning and personalization.

In sum, this study concludes that the combined effect of aerobic exercise and mindfulness-like NFB is more beneficial for task-linked ICN synchrony than the individual effect of each intervention. Combining these interventions results in more healthy executive network functioning in responders, and prevents maladaptive persistent DMN activation in non-responders, which has been associated with high levels of mind-wandering and rumination, difficulty staying on task, and dysregulated mood.

## Data Availability Statement

The raw data supporting the conclusions of this article will be made available by the authors, without undue reservation.

## Ethics Statement

The studies involving human participants were reviewed and approved by McMaster Research Ethics Board (MREB). The patients/participants provided their written informed consent to participate in this study.

## Author Contributions

SS, YL, GH, and SB conceptualized and designed the study. SS, YL, and AM ran the study and collected the data. SS developed the novel analysis pipeline with SB, and analyzed the collected data. SS and SB interpreted the data. SS wrote the manuscript in consultation with SB, JH, and MM. SB, JH, and MM provided funding for running the study. All authors contributed to the article and approved the submitted version.

## Funding

This research was supported by a Discovery Grant from the Natural Sciences and Engineering Research Council (NSERC) of Canada to SS (No. RGPIN-2019-07276), an Alexander Graham Bell Canada Graduate Scholarship (CGS-D) from NSERC Canada to SS, a Canadian Institutes of Health Research (CIHR) grant to MM (No. MWP-171647), and a NSERC Canada grant to JH (No. 296518). MM was also supported by the Homewood Chair in Mental Health and Trauma at McMaster University.

## Conflict of Interest

The authors declare that the research was conducted in the absence of any commercial or financial relationships that could be construed as a potential conflict of interest.

## Publisher's Note

All claims expressed in this article are solely those of the authors and do not necessarily represent those of their affiliated organizations, or those of the publisher, the editors and the reviewers. Any product that may be evaluated in this article, or claim that may be made by its manufacturer, is not guaranteed or endorsed by the publisher.
